# Current questions and possible controversies in autophagy

**DOI:** 10.1038/cddiscovery.2015.36

**Published:** 2015-11-09

**Authors:** L M Lindqvist, A K Simon, E H Baehrecke

**Affiliations:** 1 Cell Signalling and Cell Death Division, The Walter and Eliza Hall Institute of Medical Research, Parkville, Victoria, Australia; 2 Department of Medical Biology, The University of Melbourne, Parkville, Victoria, Australia; 3 Translational Immunology Lab, BRC-NIHR, John Radcliffe Hospital, Experimental Medicine, Nuffield Department of Medicine, Oxford, UK; 4 MRC Human Immunology Unit, Weatherall Institute of Molecular Medicine, John Radcliffe Hospital, Oxford, UK; 5 Department of Molecular, Cell and Cancer Biology, University of Massachusetts Medical School, Worcester, MA, USA

## Abstract

Interest in autophagy has exploded over the last decade, with publications highlighting crosstalk with several other cellular processes including secretion, endocytosis, and cell suicide pathways including apoptosis. Autophagy proteins have also been implicated in other cellular processes independently of their roles in autophagy, creating complexities in the interpretation of autophagy (*Atg*) mutant gene data. Interestingly, this self-eating process is a survival mechanism that can also promote cell death, but when and how autophagy may ‘switch’ its function is still under debate. Indeed, there are currently many models of how autophagy actually influences cell death. In this review, we highlight some outstanding questions and possible controversies in the autophagy field.

## Facts

- Autophagy is a cellular process that delivers cytoplasmic material to the lysosome for recycling.
- Autophagy or autophagy proteins interact with several other cellular processes such the apoptosis, secretion, and endocytic pathways.
- Autophagy proteins are involved in development and are implicated in cancer as well as neurogeneration and immune disorders.

## Open Questions

- How does the autophagy pathway interact with other pathways, such as cell suicide, secretion, and endocytic pathways?
- How common are the proposed non-canonical mechanisms of autophagy? Are there others? What is the physiological relevance of having multiple mechanisms to control autophagy?
- How far does non-canonical autophagy have to drift before it is no longer considered autophagy?
- Exactly how does the ULK1 complex, PI3K complex, and ubiquitin-like pathways communicate with each other? When some of these complexes not required, such as in non-canonical mechanisms of autophagy regulation, how is this signalling interrupted or bypassed?
- How and when do the programmed cell death and cell suicide pathways regulate autophagy? When and how does autophagy switch from facilitating cell health to promotion of programmed cell death?

**Figure 1:**
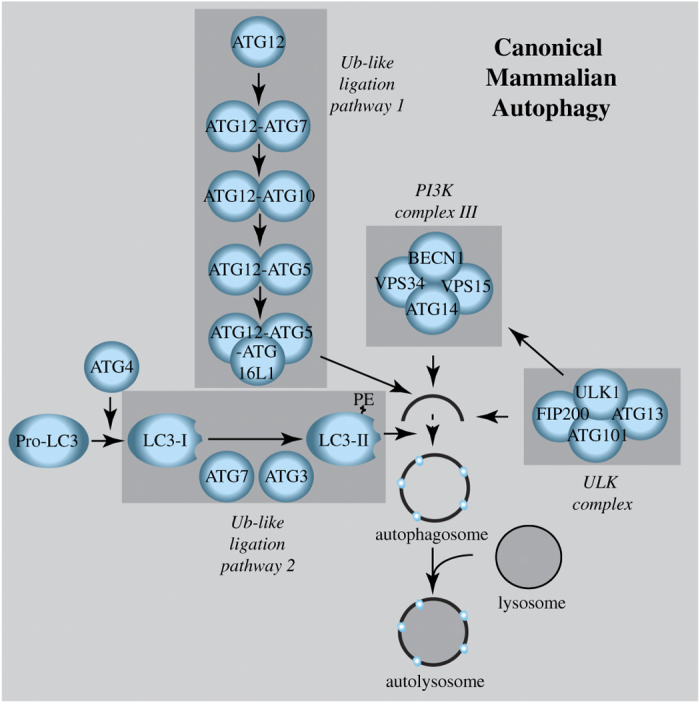
The canonical mammalian autophagy pathway. The ULK complex and the PI3K complex III initiate autophagy. When autophagy is stimulated, LC3-I is conjugated with PE by two ubiquitin-like conjugation pathways and becomes associated with the growing phagophore membrane. To degrade its contents, the autophagosome fuses with lysosomes to become autolysosomes. ATG, autophagy protein; FIP200, FAK family kinase-interacting protein of 200 kDa; Ub, ubiquitin; VPS, vacuolar protein sorting.

Autophagy, or ‘self-eating,’ is a cellular process that delivers cytoplasmic material to the lysosome for recycling. It is stimulated above the basal or resting rate when nutrients are scarce, when cells are under stress, or intracellular bacteria and damaged organelles need to be degraded. Autophagic membrane formation (i.e., the phagophore) is initiated by both the Unc-51-like autophagy-activating kinase (ULK) complex (ULK1/2(ATG1)/ FAK family kinase-interacting protein of 200 kDa (FIP200)/ATG13/ATG101) and the phosphoinositide 3-kinase (PI3K) complex III (Beclin 1 (BECN1)/VPS34/VPS15/ATG14; Figure 1),^1,2^ where ATG and VPS stand for autophagy protein and vacuolar protein sorting, respectively. Both of these complexes are major points of regulation in autophagy: both are phosphorylated by AMP-activated protein kinase (AMPK) and the mechanistic target of rapamycin (mTOR).^1,3^ The regulation of BECN1 is also discussed in more detail in question #3. When autophagy is stimulated, an ATG4-cleaved microtubule-associated protein light chain 3 (LC3) named LC3-I is conjugated with phosphatidylethanolamine (PE) to form LC3-II by an ubiquitin-like pathway, which includes ATG12, ATG7, ATG10, ATG5, and ATG16L. LC3-II is the ‘active form’ that assists in elongating the phagophore membrane, and the recruitment of cargoes to the phagophore.

Cytoplasmic material is enveloped by the phagopore to form the autophagosome. To degrade the material the autophagosome needs to fuse with lysosomes (known as the vacuole in yeast and plants), forming an autolysosome (Figure 1). This latter autophagic compartment degrades the encased cellular material and returns ‘building blocks’ such as amino acids back to the cytoplasm. While this, albeit simplified, mechanism is canonical, several alternatives have been reported, which forms our first question.

### Question #1: How is either non-canonical or canonical autophagy regulated? How can we define autophagy in experimental settings?

**Figure 2:**
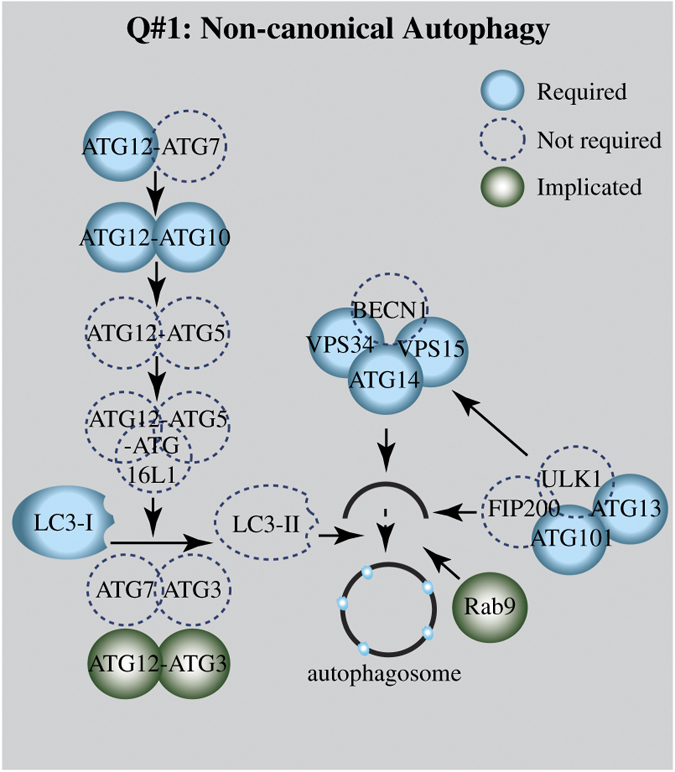
Proteins thought to be dispensable in non-canonical autophagy (question #1). Autophagy proteins that have been suggested to be dispensable for non-canonical autophagy mechanism(s) are transparent, while proteins or conjugations required only in some forms of non-canonical, but not canonical autophagy, are in green. ATG, autophagy protein; FIP200, FAK family kinase-interacting protein of 200 kDa; Rab9, Ras-related protein 9; VPS, vacuolar protein sorting.

Recent work has suggested that autophagy can occur using multiple variant mechanisms, bypassing seemingly essential complexes (Figure 2). While BECN1 is often touted as essential for autophagy, BECN1 dependency appears to be cell type specific and the protein may not be required for autophagy induced by cytotoxic compounds such as staurosporin, gossypol, or resveratrol.^4–6^

Similarly, the ULK complex is also evaded under some circumstances. Although autophagy stimulated by amino-acid starvation was ULK dependent, autophagy induced by glucose deprivation was not.^7^ Indeed, the interaction between FIP200 of the ULK complex and ATG16L1 of the ubiquitin-like LC3 lipidation pathway may not be involved in glucose-dependent autophagy.^8^ Ironically, basal or resting state autophagy appears to be ‘non-canonical’ as ATG12–ATG5 conjugation is reported to be absent. Murrow *et al.*^9^ instead argue that ATG12 conjugates with ATG3 and interestingly this also occurs when autophagy proteins are involved in endocytosis, which will be discussed further below.

But perhaps most surprising is evidence suggesting that autophagy can take place without LC3 lipidation, arguably the most common marker of autophagy. Nishida *et al.*^10^ have suggested that even though etoposide- and starvation-induced LC3B lipidation requires ATG5 and ATG7 in MEFs, the presence of double-membrane vesicles and continued protein degradation implies that autophagy occurs in the absence of these key ATG factors. They instead propose that Ras-related protein 9 (Rab9), a protein involved in trafficking between endosomes and the trans-Golgi, is involved in this non-canonical autophagy, presumably compensating for the lack of LC3-II. If this ATG5/ATG7/LC3-II-independent, but ULK1/BECN1-dependent, process is truly autophagy or if the ATG proteins are independently involved in (non-canonical) endocytosis will require further investigation (see question #2).

More recently another form of ATG7- and ATG3-independent autophagy was described during the development of the *Drosophila* midgut.^11^ In this case, genetic manipulation suggested a large panel of autophagy proteins, including ATG1, ATG2, ATG5, ATG6, ATG12, ATG13, ATG16, VPS34, and LC3 (called ATG8a in *Drosophila*), were still required for developmental autophagic cell death in these cells. Surprisingly, however, LC3 lipidation was not. The E1 ubiquitinating enzyme ubiquitin-like modifier activating enzyme 1 (Uba1) was obligatory, but does not seem to functionally replace ATG7. The exact role of Uba1 in this ATG7/3-independent autophagy will require further investigation. Importantly, autophagy stimulated by starvation in flies requires the canonical ATG7/3-dependent autophagy mechanism.^11–13^ These studies highlight that different stimuli may induce autophagy via different mechanisms in distinct cell contexts. As well, it is becoming clear that different cell types have specialised mechanisms to control autophagy, thereby allowing different responses to the same stimuli in multicellular organisms.^14^

With alternative mechanisms of autophagy proposed in which ATG proteins appear to be dispensable under some circumstances, it is easy to see how monitoring autophagy is becoming a complicated affair. No longer can we perform a simple knockdown or use a mutant to determine without reasonable doubt the role of autophagy in our experiments. These non-canonical pathways highlight our lack of knowledge of the intricacies of the molecular interactions between the autophagy complexes. How exactly does signalling take place between autophagy complexes? When some complexes are not required, how is this signalling interrupted or bypassed? What are the common forms of ‘non-canonical’ autophagy? Are they reproducible in multiple organisms under the same conditions? What is the physiological relevance/advantage of having multiple mechanisms to induce autophagy? The fact that many ATG knockout mice are embryonic lethal could suggest that alternative autophagy mechanisms are not redundant in development,^15^ although one cannot rule out that lethality is induced by additional autophagy-independent functions of these genes.

### Question #2: When does autophagy protein function become pleiotropic?

**Figure 3:**
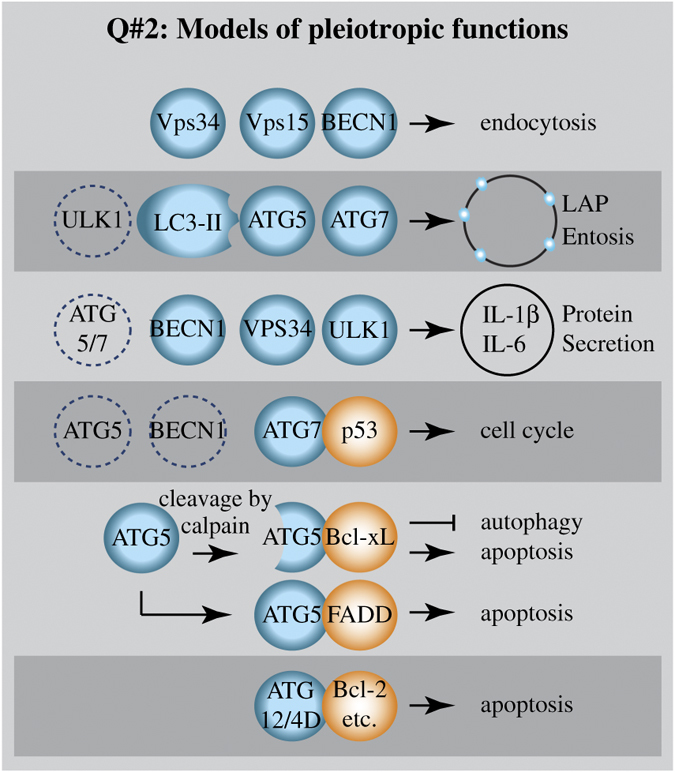
Models of pleiotropic functions of autophagy proteins (question #2). Autophagy proteins are in blue, while binding partners that function in other pathways are in orange. Proteins that are not required in these processes are transparent. ATG, autophagy protein; VPS, vacuolar protein sorting.

Autophagy proteins have also been reported to be involved in processes that are apparently independent of their roles in autophagy, further complicating data interpretation (Figure 3). For instance, the core components of the PI3K complex, including Vps34, BECN1, and Vps15 affect endocytosis, potentially at the early endosome stage.^16–19^ In addition, LC3 has been implicated in LC3-associated phagocytosis (LAP) by macrophages, which takes up foreign bodies (beads, yeast) as well as dead mammalian cells killed by multiple cell death pathways.^20,21^ This process also involves several autophagy proteins, such as BECN1, ATG5, and ATG7, but not ULK1. Loss of ATG7 caused an increase in inflammatory cytokines by macrophages, suggesting that LAPs could be anti-inflammatory, although one cannot completely rule out that inhibition of ATG7-mediated autophagy does not contribute to inflammation. Interestingly, these LAP structures are composed of a single-membrane, not double-membrane structures like classical autophagosomes. Similarly, LC3-dependent but ULK1-independent single-membrane structures have been observed during entosis, a form of cell death involving living cells that invade phagocytes to die in their phagosomes.^22^

Several autophagy proteins including BECN1, VPS34, and ULK1/2 have been reported to be involved in protein secretion.^16^ Indeed, links between autophagy (or autophagy proteins) and secretion in inflammation are mostly described in the context of an inhibitory role of autophagy in secretion of inflammatory cytokines.^23,24^ An example of an activating role is the autophagy-dependent secretion of inflammatory cytokines such as IL-1β^25^ and IL-6.^26^ As well, Atg7, but neither BECN1 nor Atg5, has been implicated in cell cycle arrest via an apparent interaction with p53.^27^ However, an Atg5- and Atg7-dependent cell cycle arrest has been observed in leukaemic cells, mediated most likely via an altered metabolism leading to increased proliferation *in vitro* and enhanced leukaemic growth *in vivo*.^28^ This topic remains controversial^29^ and requires more investigation, as it is critical for the understanding of autophagy’s role in cancer. In addition, sequestosome-1 (SQSTM1/p62), a protein that binds ubiquitinated protein aggregates for delivery to autophagosomes, binds to kelch-like ECH-associated protein 1 (Keap1). This displaces and activates the transcription factor nuclear factor (erythroid-derived 2)-like 2 (Nrf2) thereby helping to protect against oxidative and electrophilic stresses.^30^ Therefore, a decrease in autophagy would induce SQSTM1/p62 accumulation, thereby regulating this interaction. Several other apparent functions of autophagy proteins have been recently reviewed.^31^

One question that arises is if these apparent pleiotropic ATG functions are either actually independent of autophagy, or if autophagy is simply non-canonical (see question #1). Although most studies demonstrated that one or two other autophagy proteins are not required, they rarely systematically study the role of a large panel of the autophagy protein-encoding genes. For these types of studies, it is frustrating that we do not yet have specific autophagy inhibitors – chloroquine and bafiolomycin A1 influence the lysosome and therefore impact the trafficking processes that intersect with the lysosomal network.^1,32,33^ By contrast, 3-methyladenine and wortmannin target multiple PI3Ks including the known pleiotropic Vps34, and only recently have more specific Vps34 and ULK1 inhibitors been reported.^34–36^ Clearly, even with definitive evidence, we must face the philosophical question: if only part of the pathway is required for delivery of cargoes to lysosomes, is it considered autophagy dependent or independent? When does a vesicle made by autophagy proteins become sufficiently different that the functions of the proteins are considered pleiotropic? Perhaps clearer definitions are needed, but if the original definition of autophagy includes any mechanism enabling delivery of materials to the lysosome, then lysosome-independent functions of ATG genes may need to be defined with new names.

Several autophagy proteins have also been implicated in cell death. A calpain-cleaved fragment of ATG5, which is no longer functional in autophagy, induced cell death by apparently sequestering the pro-survival protein B-cell lymphoma-extra large (Bcl-xL), thereby inducing intrinsic apoptosis.^37^ As well, ATG5 has been reported to interact with Fas-associated protein with death domain (FADD) to regulate caspase 8-dependent apoptosis.^38^

ATG12 has been suggested to induce apoptosis by sequestering the pro-survival B-cell lymphoma 2 (Bcl-2) family members via its BH3 domain – a domain common to the apoptotic proteins such as Bim.^39^ It is interesting to note that ATG4D and BECN1 also have a BH3-like domain,^40,41^ although one would predict that both would have a lower affinity binding to Bcl-2 or Bcl-xL compared with the apoptotic family members. Consistent with this prediction, overexpression of ATG4D stimulated mitochondrial apoptosis, which was not strictly dependent on its BH3 domain.^40^ As well, apoptotic family members have been implicated in regulating autophagy by binding to BH3 domains, which leads to our next question.

### Question #3: How do cell death pathways regulate autophagy?

**Figure 4:**
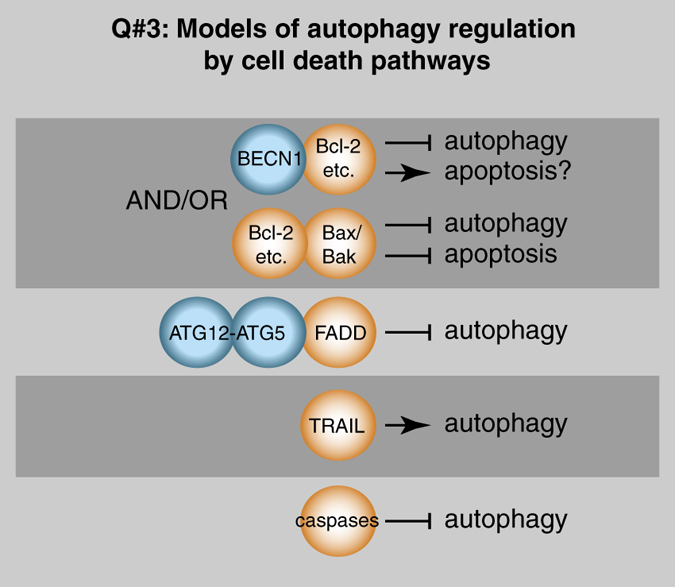
Models of cell death pathways regulating autophagy (question #3). Autophagy proteins are coloured in blue, while the proteins involved in apoptosis they are proposed to interact with are coloured orange. ATG, autophagy protein.

The most investigated crosstalk in the cell death and autophagy regulatory pathways is between the pro-survival Bcl-2 family members and BECN1.^42,43^ Multiple models exist for this relationship (Figure 4). The original model has Bcl-2, and its related family members myeloid cell leukaemia 1 (Mcl-1) and Bcl-xL, binding to and inhibiting BECN1 independently of the apoptosis pathway.^42,44^ Mutagenesis and crystallography data indicate that this interaction occurs via a BH3-like domain on BECN1.^41^ As the binding affinity of BECN1 appears to be approximately a thousand-fold weaker than that of the canonical apoptotic BH3-containing proteins, such as either Bim or Bcl-2 homologous antagonist killer (Bak),^41,45–47^ it will be interesting to study if and how these interactions regulate each other in the cell. BECN1 is unusual in that overexpression did not appear to induce apoptosis like other proteins with BH3 domains.^42^ On the other hand, it has also been reported that phosphorylation of BECN1 by hepatocyte growth factor-like protein (Mst1) can in fact increase the affinity of BECN1 for Bcl-2, thereby supressing autophagy but also importantly inducing apoptosis.^48^

To complicate matters, recent evidence suggests that modulating the pro-survival Bcl-2 family only influenced autophagy when Bcl-2-associated X protein (Bax) and/or Bak were present. This led to the proposal of an alternative model where the pro-survival Bcl-2 family members inhibit autophagy by restraining their apoptotic interaction partners Bax and Bak, the effectors of intrinsic apoptosis.^43,49^ Consistent with this model, overexpression of Bax alone can stimulate autophagy.^50^ Others, however, report the opposite, presumably owing to later downstream caspase cleavage of autophagy proteins.^51,52^ Further research will be needed to determine whether these two models are mutually exclusive, can occur in tandem, or whether they are specific to circumstance. For instance, overexpressing Bcl-2 did not inhibit either LC3 lipidation or autophagosome formation during autophagy induced by detachment or growth factor withdrawal.^53^ However, it appears that Bax/Bak-independent effects of inhibiting the pro-survival Bcl-2 family may be possible, but they occur much later than autophagy instigated by Bax and Bak, which is induced at a similar rate to starvation-induced autophagy.^43,54^

In addition, several other cell death proteins have been suggested to affect autophagy (Figure 2c).^55^ FADD, which is involved in extrinsic apoptosis, has been reported to regulate autophagy by binding to ATG5-ATG12.^38,56^ TNF-related apoptosis-inducing ligand (TRAIL), a ligand that can induce extrinsic apoptosis and necroptosis, has been suggested to activate autophagy in MCF-10A cells, independent of caspase activity.^57^ However, caspases have also been suggested to inhibit autophagy.^58^ In the latter study, the caspase inhibitor Z-VAD-FMK and caspase 8 inhibition have both been proposed to inhibit RIP-dependent autophagy and cell death in L929 cells. It was therefore suggested to be autophagic cell death, but with our current knowledge of necroptosis (a RIP-dependent cell death induced by caspase inhibition),^59^ another interpretation could be that this is actually necroptotic cell death with accompanying autophagy. Indeed, this forms a transition to our last topic – autophagic cell death.

### Question #4: When is autophagy either a pro-survival or a cell death process?

**Figure 5:**
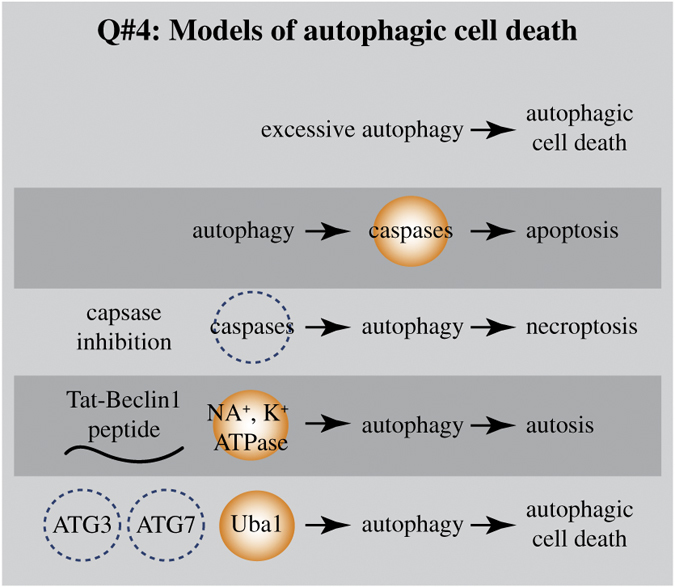
Proposed autophagic cell death mechanisms (question #4). Proteins shown that are not required for this process are transparent. ATG, autophagy protein.

Autophagic cell death has been a controversy for quite some time (Figure 5).^60^ Initially it was defined as cell death that correlated with stimulation of autophagy, but the current and accepted definition of autophagic cell death is ‘a cell death subroutine that is limited or delayed by the pharmacologic or genetic inhibition of the autophagic machinery.’^61^ This is an important distinction as apoptosis, necroptosis, and necrosis are frequently accompanied by autophagic vacuoles. An example is the induction of cell death by cytotoxic compounds. For instance, while it is clear that the apoptosis-inducing chemotherapeutic etoposide can induce autophagy independently of apoptosis (i.e., absence of the effectors Bax and Bak),^43,62^ the manner of death induced when apoptosis is inhibited is still under dispute. Although cell death was inhibited with autophagy inhibitor 3-methyladenine, which inhibits multiple PI3Ks,^62^ genetic inhibition of autophagy did not affect viability.^43^ In a screen of 1400 cytotoxic compounds, none induced cell death in an ATG7-dependent manner even though knockdown of ATG7 was able to reduce autophagosome formation.^63^ However, taking into account question #1, there is the possibility that autophagic cell death occurred in an ATG7- and LC3-independent manner. There is some controversy regarding the role of autophagy in luminal formation at least *in vitro*. For instance, TRAIL-induced autophagy has been suggested to both contribute to cell death and to promote cell survival to induce luminal filling.^53,57,64^

A subtype of autophagic cell death called autosis has recently been described. When an 18-amino-acid fragment of BECN1 was fused to a HIV Tat peptide and fed to MEF and HeLa cells, autophagy and cell death occurred, which was independent of loss of Bax and Bak (apoptosis) or RIPK1 and RIPK3 (necroptosis).^65^ Na^+^, K^+^-ATPase appears to contribute to this type of cell death, although why this type of Na^+^, K^+^-ATPase would either be activated by autophagy or stimulate cell death is unclear. Although Tat-BECN1 peptide was used to characterise autosis the majority of the time, 1% of starved HeLa cells also possess this phenotype. It would be interesting to determine whether the same type of cell death occurs in starved cells that are incapable of undergoing apoptosis or necrosis. Autosis also occurred after hypoxic–ischaemic injury in neonatal rat neurons, but it is unclear how frequently autosis occurs *in vivo*. Under what stresses is it activated? Is this form of cell death also present when other cell death pathways are genetically inactivated *in vivo*? This publication raises several interesting questions and leads to the query whether autosis is actually different enough from autophagic cell death (cell death caused by autophagy) to justify a separate name.^66,67^

Perhaps the purest approach to determining whether autophagy contributes to cell killing is to ask this question in the context of programmed cell deaths that occur *in vivo* during development. During *Drosophila* development, programmed cell death removes the larval midgut, which is genetically independent of caspase-mediated apoptosis.^68^ This cell death was inhibited by the deletion of autophagy genes. Interestingly, this seems to be a type of non-canonical autophagy, which does not require ATG7 or ATG3.^11^ Is autophagy associated with cell death induced via a different mechanism than autophagy that is pro-survival? Strong data exist in support of this possibility in the fly salivary gland, where immune receptor signalling, micro RNA, and calcium signalling have been shown to be required for autophagy and cell death, but these genes are not required for nutrient deprivation-induced autophagy and cell survival in the fly fatbody.^69,70^ Alternatively, does autophagy in the context of cell survival and death possess different feedback signalling mechanisms? Another possibility is that different autophagic cargoes are recruited during autophagy associated with cell death than during cell survival.^71,72^ Although autophagic cell death is quite well characterised in the fly, the question remains whether this type is limited to the fly or whether cells from other organisms undergo programmed autophagic cell death in a similar manner. Indeed, several autophagy genes are required for development in the mouse as well,^15^ but whether this is as a programmed cell death function or to keep cells alive has not been determined. Autophagic cell death in other organisms, such as other insects, protists, and plants, is discussed in detail in Nelson *et al.*^73^

To complicate matters further, autophagy is reported to kill via other cell death pathways as well. For instance, several studies have suggested that autophagy can induce cell death via mitochondrial-mediated apoptosis.^39,74,75^ In addition, a recent report suggests that autophagy stimulates necroptosis after certain stimuli^76^ and this may be consistent with previous work,^58^ but how this occurs is not clear. It would be interesting to determine whether autophagy stimulates necroptosis after ‘classical’ necroptosis stimuli (TNF, cycloheximide, and caspase inhibition) in an MLKL-dependent manner. Obviously there are still many outstanding questions regarding autophagic cell death. When and how does autophagy ‘switch’ from pro-survival to become a cell death process or programmed cell death mechanism? Is this environment specific, organism specific or tissue specific? What pathway(s) ‘create the switch’ if there is one? Why is it different in distinct cell types? When autophagy kills, how are certain mechanisms such as autosis or caspase-dependent cell death activated? Can autophagy also kill by necrosis?

## Concluding Remarks

Here we have highlighted a small number of the interesting current questions in the autophagy field. Many questions exist about the fundamental mechanisms that control this important process,^77^ and differences in cell and tissue biology in multicellular animals require robust genetic investigation of this process in model systems.^14^ Although efficient and economical, the initial characterisation of cell death and autophagy mechanisms in cell lines has the disadvantage of potentially not being physiologically relevant, especially if work is done in cell lines that have large chromosomal deletions that include pertinent autophagy and apoptosis genes. We now need to focus on finding *in vivo* evidence for these mechanisms and determining their physiological role, including for non-canonical autophagy.

The autophagy field is in an exciting time. The more we understand about the intricacies of the pathway, its variant mechanisms, and its interactions with other pathways, the closer we will get to translate our knowledge to the clinic. Autophagy has been considered a promising target for disease therapies.^78,79^ Patients with cancer, neurogeneration, or immune disorders may one day benefit from our increased understanding of the interplay between autophagy and cell death and alternative autophagy pathways. Undoubtedly many of our current models and definitions will be modified or even discarded as our understanding of this complex pathway and its interactions grows. Our different points of view and scientific debate help to foster new ideas and allow science to progress. Indeed, ‘all great ideas are controversial, or have been at one time.’ – Gilbert Seldes.

## Abbreviations

AMPK: AMP-activated protein kinase
ATG: autophagy protein/gene
Bak: Bcl-2 homologous antagonist killer
Bax: Bcl-2-associated X protein
Bcl-2: B-cell lymphoma 2
Bcl-xL: B-cell lymphoma-extra large
BECN1: Beclin 1
FADD: Fas-associated protein with death domain
FIP200: FAK family kinase-interacting protein of 200 kDa
Keap1: kelch-like ECH-associated protein 1
LAP: LC3-associated phagocytosis
LC3: microtubule-associated protein light chain 3
Mcl-1: myeloid cell leukaemia 1
mTOR: mechanistic target of rapamycin
Nrf2: nuclear factor (erythroid-derived 2)-like 2
PE: phosphatidylethanolamine
Rab1: Ras-related protein 9
SQSTM1: sequestosome-1
TRAIL: TNF-related apoptosis-inducing ligand
Uba1: ubiquitin-like modifier activating enzyme 1
ULK: Unc-51-like autophagy-activating kinase
VPS: vacuolar protein sorting
